# Association of Borderline Intellectual Functioning and Adverse Childhood Experience with adult psychiatric morbidity. Findings from a British birth cohort

**DOI:** 10.1186/s12888-019-2376-0

**Published:** 2019-12-05

**Authors:** Angela Hassiotis, Emma Brown, James Harris, David Helm, Kerim Munir, Luis Salvador-Carulla, Marco Bertelli, Amaria Baghdadli, Jannelien Wieland, Ramon Novell-Alsina, Jordi Cid, Laura Vergés, Rafael Martínez-Leal, Tuba Mutluer, Fuad Ismayilov, Eric Emerson

**Affiliations:** 1 Division of Psychiatry, University College London, 149 Tottenham Court Road, London, W1T 7NF UK; 2 Camden & Islington Foundation Trust, St Pancras Hospital, London, UK; 3 Developmental Neuropsychiatry, Department of Psychiatry and Behavioral Sciences, Bloomberg Children’s Center, The Johns Hopkins Hospital, Baltimore, MD USA; 4 Institute for Community Inclusion, Division of Developmental Medicine, Boston Children’s Hospital, Boston, MA USA; 5 Developmental Medicine Center, Division of Developmental Medicine, Boston Children’s Hospital and Harvard Medical School, Boston, MA USA; 6 Centre for Mental Health Research, Australian National University, Acton, Australia; 7 CREA, Research and Clinical Centre, San Sebastiano Foundation, Florence, Italy; 8 Child and Adolescent Psychiatry Department, Montpellier Hospital University, Montpellier, France; 9 Kristal Centre for Psychiatry and Intellectual Disability, Rivierduinen, Leiden, The Netherlands; 10 Mental Health and Intellectual Disability Specialized Service, Catalan Health Govenment. Martí i Julià Hospital, Girona, Spain; 11 Intellectual Disability and Developmental Disorders Research Unit (UNIVIDD), Fundació Villablanca, IISPV, Universitat Rovira i Virgili, CIBERSAM, Reus, Spain; 12 Department of Child and Adolescent Psychiatry, Koc University Hospital, Istanbul, Turkey; 13 Department of Psychiatry, Azerbaijan Medical University, Baku, Azerbaijan; 14 Centre for Disability Research & Policy, Faculty of Health Sciences, University of Sydney, Sydney, Australia

**Keywords:** Borderline, Intellectual, Adversity, Mental wellbeing, Childhood

## Abstract

**Background:**

To examine whether Borderline Intellectual Functioning (BIF) and Adverse Childhood Experiences independently predict adult psychiatric morbidity.

**Methods:**

We performed a secondary analysis of longitudinal data derived from the 1970 British Birth Cohort Study to examine whether BIF and Adverse Childhood Experiences independently predict adult mental distress as measured by the Malaise Inventory. Factor analysis was used to derive a proxy measure of IQ from cognitive testing at age 10 or 5. Variables that could be indicators of exposure to Adverse Childhood Experiences were identified and grouped into health related and socio-economic related adversity.

**Results:**

Children with BIF were significantly more likely than their peers to have been exposed to Adverse Childhood Experiences (BIF mean 5.90, non-BIF mean 3.19; Mann-Whitney z = 31.74, *p* < 0.001). As adults, participants with BIF were significantly more likely to score above the cut-off on the Malaise Inventory. We found statistically significant relationships between the number of socio-economic Adverse Childhood Experiences and poorer adult psychiatric morbidity (r range 0.104–0.141, all *p* < 001). At all ages the indirect mediating effects of Adverse Childhood Experiences were significantly related to adult psychiatric morbidity.

**Conclusions:**

The relationship between BIF and adult psychiatric morbidity appears to be partially mediated by exposure to Adverse Childhood Experiences. Where possible, targeting Adverse Childhood Experiences through early detection, prevention and interventions may improve psychiatric morbidity in this population group.

## Background

Exposure to Adverse Childhood Experiences (ACEs) represents a significant threat to the health and wellbeing of children. A broad definition of ACEs encompasses events occurring in a child’s family or social environment that may cause harm or distress. This includes proximal traumatic events (e.g. physical, verbal or sexual abuse) and problematic aspects of family functioning (e.g. parental domestic violence, substance abuse or separation, household poverty). ACEs impact a great number of the general population, with evidence suggesting that 57% of children have experienced one or more ACEs [1].

Exposure to ACEs has been associated with negative health consequences in later life, such as diabetes, cardiovascular problems and cancer [2–4] and is also thought to impact adult mental health. Hughes et al. [1] showed that those who have experienced four or more ACEs were around four times more likely to experience adult mental distress. Those who have experienced ACEs have increased rates of psychiatric disorders, including depression, anxiety, Post Traumatic Stress Disorder (PTSD), schizophrenia, substance dependence, self-harm and suicide attempts [5–7]. This relationship has been replicated across high, middle and low income countries around the world [8]. More specifically, there is strong evidence indicating a link between low socioeconomic status in childhood and adult mental health, with the type and duration of socioeconomic adversity in childhood predicting the onset, duration and severity of adult mental health disorders [8–10].

Research has shown that children with intellectual impairments are at greater risk of being exposed to ACEs compared with their peers. Children with intellectual disability are between three and seven times more likely to experience neglect, physical, emotional and sexual abuse and experience a broader range of ACEs compared to other children [11–13]. They also have higher rates of mental health problems across the lifespan [14–17].

People labelled with Borderline intellectual functioning (BIF) typically score lower on tests of intellectual ability and other indices of cognitive functioning than the general population, but not to the extent to be defined as an intellectual disability. BIF does not present with a specific symptom or set of symptoms but as a continuum of risk across the lifespan. The closer the proximity to Intellectual Disability, or greater the degree of cognitive and adaptive impairments, the higher the likelihood of associated risks that need to be monitored throughout development in order to encourage better coping strategies and healthy behaviours.

When considering Intelligence Quotient (IQ), BIF is commonly defined as between one and two standard deviations below the mean on standardised tests of intelligence, (IQ range 70–85). Therefore, according to the normally distributed curve of IQ, the range spans the 2nd to the 16th percentile, including 14% of the population [18]. It is not clear whether BIF should be regarded as a risk factor, as a dimensional health condition or as a category within the developmental disorders. BIF was not included in DSM-5 [19] as a neurodevelopmental disorder (F code) diagnosis but it is listed in both DSM-5 and the US ICD-10-CM [20] among “Other Conditions That May Be a Focus of Clinical Attention” (R41.83).

Policy changes and societal attitudes towards those with intellectual impairments have contributed to lack of awareness and neglect of people with BIF. It often goes unrecognised by health or social care professionals, who do not receive routine training in the identification of BIF or in adapting interventions to address their cognitive needs, resulting in inappropriate or lack of formal support [21, 22]. The lack of attention given to BIF persists not only through professional services but through research, with comparatively little interest in investigating this group [23].

Despite the lack of clarity around definition and recognition, individuals with BIF as a group are particularly at risk. The literature shows that adults with BIF have higher rates of incarceration, job insecurity, drug use and poor social functioning and are over twice as likely to have a psychiatric diagnosis as people with an average or above IQ [24–26]. This association has been found for depression, anxiety disorders, post-traumatic stress disorder, psychosis, substance abuse, personality disorders, self reported suicide attempts and neurodevelopmental disorders [27–33].

There is indication that those with BIF are also at greater risk of early life stressors. Children with BIF are more likely to experience bullying, poor parenting, poverty, material hardship and parental unemployment than typically developing children [34–37]. Interestingly, Emerson et al. [37] replicated findings that children with BIF had an increased risk of mental health problems, but found that when the confounding effect of exposure to socio-economic disadvantage was controlled for, the higher prevalence of mental health problems was greatly reduced. This prompts questions regarding the mediating effect that childhood experiences may have on the mental health of adults with BIF.

Most published studies of the impact of ACE on children or adults use cross-sectional samples. In the current paper we used a longitudinal cohort from the UK to investigate 1) the association between BIF and adult psychiatric morbidity; 2) the association between BIF and exposure to ACEs; 3) the extent to which exposure to ACEs moderates and/or mediates the association between BIF and adult psychiatric morbidity. We hypothesised that children with BIF would have greater exposure to ACEs than their typically developing peers, and that this exposure to ACEs would partially mediate the higher rates of adult psychiatric morbidity.

## Method

The study is based on a secondary analysis of data from six waves of the 1970 British Cohort Study (BCS70). BCS70 is following up over 17,000 children born during one week in the UK in 1970, with data collected soon after birth (first wave) [38, 39] . Since then, additional waves have taken place in at age 5 (*n* = 12,939), 10 (*n* = 14,350), 16 (*n* = 11,206), 26 (*n* = 8654), 30 (*n* = 10,833), 34 (*n* = 9316), 38 (*n* = 8874) and 42 (*n* = 9717) years [40–42]. The surveys cover health status; health behaviours; wellbeing; educational attainment; employment and occupation; financial status; social and civic participation; social support; family formation and crime. Anonymised data from ages 5, 10, 26, 30, 34 and 42 follow-up surveys were downloaded from the UK Data Service for this study [43–48].

### Identifying participants with borderline intellectual functioning

While BCS70 includes measures of child cognitive functioning at ages 5 and 10 [49], these were not validated IQ tests but instead, a range of brief tests drawn from existing IQ tests were administered, or tests of assessed attainment that is likely to be related to IQ. In this study, we have followed established procedures in deriving a proxy [49–51].

At age 10, eight tests were administered: the Shortened Edinburgh Reading Test [52]; the Friendly Maths Test [49]; the Pictorial Language Comprehension Test [49]; the Spelling Dictation task [49]; and four subscales of the British Ability Scales, Word Definitions, Word Similarities, Recall of Digits and Matrices [53]. In total, 12,885 (87%) of children participating at age 10 completed at least one assessment; 11,134 (75%) children completed all assessments [49].

Cognitive test results at age 5 were available for an additional 2568 children who, however, did not have test results reported at age 10. At age 5, the children were administered: the Copying Designs Test [54]; the English Picture Vocabulary Test [55]; the Human Figure Drawing (Draw-a-Person) Test [56]; the Complete a Profile Test [57]; and the Schonell Reading Test [58]. In total, 13,059 (99%) of all children participating in the age 5 survey completed at least one assessment and 11,254 (86%) children completed all assessments [49].

### Childhood adversities

Data collected at age 5 and age 10 follow-ups were reviewed to identify variables that indicated exposure to low socio-economic position and specific adverse childhood experiences as included in a recent systematic review [1]. We identified 25 variables (13 at age 10, 12 at age 5). Of these, 19 were based on 11 indicators of social and/or material deprivation (living in a poor area, living in damp housing, living in overcrowded housing, living in rented housing, low parental educational attainment, low social class, low household assets, low income, living in a workless household, living in a single parent family, potential maternal psychiatric morbidity). All but three of these (living in damp housing, living in overcrowded housing, low income) were collected at ages 5 and 10. The remaining six indicators related to three health-related adversities (accident requiring medical treatment, hospital out-patient attendance at ages, hospital in-patient admission) each collected at ages 5 and 10. Initial inspection of these data indicated that the indicators based on social and/or material deprivation performed well as a simple additive scale (alpha = 0.80), but inclusion of the indicators based on health-related adversities significantly reduced the scale’s internal consistency. As a result, we created a separate additive scale for health related adversities (alpha = 0.53).

### Adult psychiatric morbidity

At ages 26 and 30 the 24-item self-completed *Malaise Inventory* was used to measure levels of anxiety and/or depression, with potential adult psychiatric morbidity being identified by a score of eight or more [59]. At ages 34 and 42 an abbreviated 9-item version of the Malaise Inventory was used, with a potential mental health problem being identified by a score of four or more.

### Statistical analysis

In order to maximise use of participants’ data and to reduce potential bias resulting from exclusion of partial non-respondents, missing cognitive test data for partial respondents were imputed using multiple imputation routines in IBM SPSS 22. Five parallel data sets were imputed and then averaged to create the final imputed data.

Principal components analysis was used to establish the presence of a general cognitive ability factor across tests and standardised scores on the first component were extracted as a proxy indicator for IQ [49–51]. At age 10, the first extracted component accounted for 59% of the variance of initial eigenvalues with all tests loading positively on the component (loading range 0.55–0.88). For the respondents with missing data on cognitive testing at age 5 we followed the procedures outlined above to: (1) impute partially missing cognitive test results; (2) establish the presence of a general cognitive ability factor across tests (*g*); (3) use standardised scores on *g* as an indicator of IQ at age 5. At age 5, the first extracted component accounted for 41% of the variance of initial eigenvalues with all tests loading positively on the component (loading range 0.47–0.76).

Exploratory analyses indicated significantly higher attrition rates among participants with BIF than those without BIF. We addressed attrition by imputing missing data (arising from either wave or item non-response) as previous analyses of BCS70 had indicated that imputation models were preferable to the use of sample weights [60]. We used sex, BIF status, child behaviour problems at ages 5 and 10 and available responses to the Malaise Inventory at ages 26, 30, 34 and 42 to impute missing adult mental health data.

Firstly, we used simple descriptive statistics to characterise the associations between: (1) BIF and exposure to childhood adversities; and (2) exposure to childhood adversities and adult mental health.

Second, we estimated the strength of association between BIF and adult mental health in four models. In Model 1 we reported unadjusted prevalence rate ratios (with 95% confidence intervals) for adult mental health problems among participants with BIF (participants without BIF being the reference group). In Model 2 we estimated prevalence rate ratios for adult mental health problems among participants with BIF adjusted for between group differences in participant gender as initial exploratory analysis indicated that BIF was more common among males (14.3% vs. 12.9%). In Model 3 we estimated prevalence rate ratios for adult mental health problems among participants with BIF adjusted for between group differences in participant gender and exposure to childhood adversities. For these analyses we recoded the number of adversities into population-based quintiles. In Model 4, we included BIF*childhood adversities interaction terms into the model to estimate whether BIF status moderated the association between exposure to childhood adversities and adult mental health problems. Prevalence rate ratios were estimated using Poisson regression with robust standard errors [61, 62] using fully imputed datasets and datasets in which missing data on adult mental health outcomes were not imputed. We primarily report analyses using fully imputed datasets, with comments on any notable variation between analyses conducted with the imputed and non-imputed data.

## Results

Of the 15,453 participants, 426 (2.8%) were functioning in the IQ range associated with intellectual disability (IQ 70 or below), 2108 (13.6%) were functioning in the BIF range (IQ range 71–85) and 12,919 (83.6%) were functioning in a higher IQ range (IQ 86+).

**Fig. 1:**
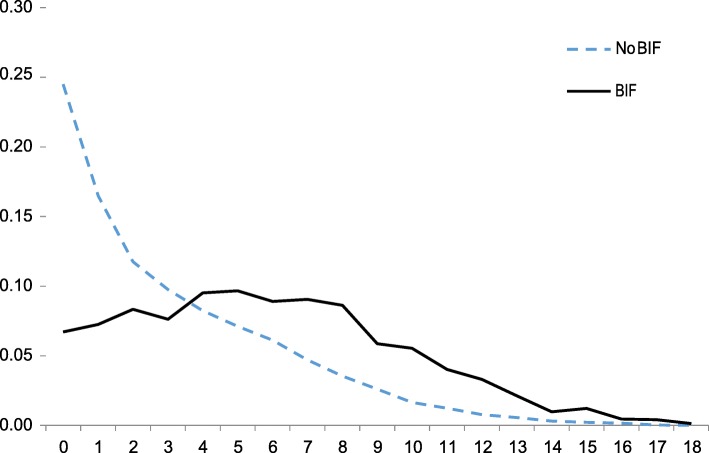
Proportional frequency distribution of exposure to ACEs for children with and without borderline intellectual functioning

Children with BIF were significantly more likely than their peers to have experienced childhood adversities related to social and/or material deprivation (number of adversities: BIF mean 5.90 median 6; non-BIF mean 3.19 median 2, Mann-Whitney z = 31.74, *p* < 0.001) and childhood adversities related to health events (number of adversities: BIF mean 2.45 median 2, non-BIF mean 2.15 median 2, Mann-Whitney z = 7.14, *p* < 0.001). The proportional frequency distribution of exposure to adversities related to social and/or material deprivation is shown in Fig. 1.

**Fig. 2:**
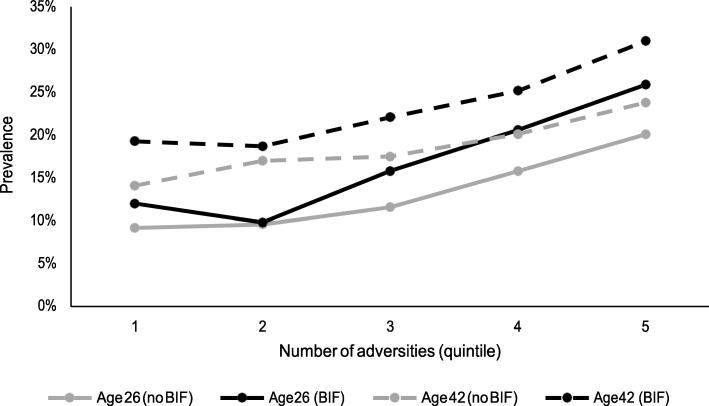
Association between exposure to childhood adversities associated with material and/or social deprivation and adult psychiatric morbidity at ages 26 and 42 for participants with and without BIF

At all adult ages there were statistically significant relationships between the number of socio-economic adversities exposed to in childhood and higher Malaise Inventory scores for participants with BIF (Spearman’s r range 0.09–0.14, all *p* < 0.001) and without BIF (Spearman’s r range 0.08–0.12, all *p* < 0.001). Example data are provided in Fig. 2. For participants with BIF there were no statistically significant relationships between the number of health adversities in childhood and higher adult Malaise Inventory scores at any age. For participants without BIF only at age 34 was there a statistically significant relationships between the number of health adversities in childhood and higher adult Malaise Inventory scores (r = 0.02, *p* < 0.05).

Unadjusted prevalence rates and unadjusted and adjusted prevalence rate ratios are presented in Table 1 for fully imputed data and in Table 2 for data in which only the predictor variables were imputed. In the fully imputed data there were highly significant differences in unadjusted prevalence rate ratios for possible adult psychiatric morbidity between participants with and without BIF at all ages. Adjusting these ratios for between-group differences in gender (Model 2) marginally increased the prevalence rate ratios at all ages. Further adjusting these ratios for between-group differences in exposure to childhood adversities (Model 3) significantly reduced the prevalence rate ratios at all ages. The percentage reduction in risk evident for possible adult psychiatric morbidity among participants with BIF in Model 3 raged from 51% at age 26 to 24% at age 42. Risk attenuation in Model 3 reflected the increased risk of participants with BIF being exposed to childhood adversities associated with material and/or social deprivation. Exposure to health-related adversities did not significantly contribute to Model 3 results at any age. Assuming Type 1 and Type 2 error rates of 0.05, the sample size of participants with BIF is greater than needed to detect a correlation coefficient greater than 0.08 and the sample size of participants without BIF is greater than needed to detect a correlation coefficient greater than 0.04. There was no evidence of effect modification of exposure to childhood adversities associated with material and/or social deprivation by BIF status at any age.

**Table 1:** Prevalence and prevalence rate ratios of potential mental health problems. in adulthood by borderline intellectual functioning status and age (fully imputed data**).**

	Age 26	Age 30	Age 34	Age 42
Borderline intellectual functioning prevalence (with 95% CI)	20.7% (17.1–24.8%)	20.8% (17.2–24.9%)	23.5% (19.7–27.8%)	26.2% (22.2–30.6%)
Higher estimated IQ prevalence (with 95% CI)	12.9% (12.3–13.5%)	11.5% (11.0–12.1%)	15.0% (14.4–15.6%)	18.2% (17.5–18.9%)
Model 1: Unadjusted prevalence rate ratio (with 95% CI)	1.60 (1.45–1.76)	1.81 (1.64–1.99)	1.57 (1.44–1.71)	1.45 (1.33–1.56)
*P* value	< 0.001	< 0.001	< 0.001	< 0.001
Model 2: Prevalence rate ratio adjusted for participant gender	1.63 (1.48–1.79)	1.83 (1.67–2.02)	1.64 (1.50–1.78)	1.46 (1.35–1.58)
P value	< 0.001	< 0.001	< 0.001	< 0.001
Model 3: Prevalence rate ratio adjusted for participant gender and exposure to childhood adversities	1.31 (1.19–1.45)	1.53 (1.38–1.69)	1.35 (1.23–1.47)	1.35 (1.25–1.47)
P value	< 0.001	< 0.001	< 0.001	< 0.001
Model 4: Significance of BIF status by childhood adversities associated with material and/or social deprivation	n.s	n.s	n.s	n.s

**Table 2:** Prevalence and prevalence rate ratios of potential mental health problems.in adulthood by borderline intellectual functioning status and age (only childhood adversity imputed).

	Age 26	Age 30	Age 34	Age 42
Borderline intellectual functioning prevalence (with 95% CI)	17.0% (14.5–19.8%)	16.7% (14.5–19.8%)	19.6% (17.2–22.2%)	22.0% (19.3–25.0%)
Higher estimated IQ prevalence (with 95% CI)	12.7% (11.9–13.5%)	11.6% (10.9–12.3%)	14.4% (13.6–15.2%)	17.6% (16.6–18.5%)
Model 1: Unadjusted prevalence rate ratio (with 95% CI)	1.34 (1.13–1.59)	1.44 (1.25–1.66)	1.36 (1.18–1.57)	1.26 (1.09–1.45)
P value	< 0.01	< 0.001	< 0.001	< 0.01
Model 2: Prevalence rate ratio adjusted for participant gender	1.37 (1.16–1.61)	1.45 (1.26–1.68)	1.38 (1.20–1.59)	1.26 (1.09–1.45)
P value	< 0.001	< 0.001	< 0.001	< 0.01
Model 3: Prevalence rate ratio adjusted for participant gender and exposure to childhood adversities	1.12 (0.94–1.32)	1.21 (1.04–1.40)	1.17 (1.01–1.35)	1.10 (0.95–1.27)
P value	n.s.	< 0.05	< 0.05	n.s.
Model 4: Significance of BIF status by childhood adversities associated with material and/or social deprivation	n.s	n.s	n.s	n.s

Notes: *CI* = Confidence interval, *n.s*. = not significant at alpha *p* < 0.05

Analyses which did not involve the imputation of dependent variables showed a very similar pattern (Table 2). The key differences are: (1) notable lower prevalence estimates for possible adult mental health problems, especially among participants with BIF; (2) notable lower prevalence rate ratios for possible adult mental health problems among participants with BIF at all ages; (3) Model 3 adjustments reducing the statistical significance of increased risk for possible adult mental health problems among participants with BIF to non-significant levels at ages 26 and 42.

## Discussion

This study is the first, to our knowledge, to take a longitudinal approach in examining the association between BIF and adult psychiatric morbidity and its association with ACEs. It is the strongest evidence yet that children with BIF are at greater risk of exposure to ACEs than their peers.

More work needs to be done to fully clarify the causal mechanisms underlying the association between BIF and ACEs. Previous research by Chen et al. reported higher mental health problems among children with IQ scores between 70 and 80 (in the BIF range) compared to those with IQ scores below 70 [63]. This finding was consistent with prior concept that the BIF group may be sensitive enough to understand age or role appropriate expectations and may be frustrated by not being able to reach these levels without recourse to professional support for which they may not be eligible [64].

The long-term effects of ACEs may also exacerbate cognitive sets and shape negative styles of interpersonal interactions and relationships [65]. Another consideration is that effects of ACE in early life may lead to functional and structural changes in the neuroendocrine system [66] with consequent impact on mental health. A counter-point is that coexistence of BIF, ACEs and adult psychiatric morbidity implies that the association does not represent environmentally mediated risk processes since risk experiences are not randomly distributed and may, in part, reflect genetic mediation [67].

### Strengths and limitations

This is the largest population-based study to examine associations between BIF, ACEs and adult psychiatric morbidity. It demonstrates, using a longitudinal analysis, that effects of ACEs are exaggerated in individuals with BIF and that their coexistence may play a contributory role in the onset of adult mental disorders.

However, our study has a number of limitations. First, the interpretation of the findings is hindered by the use of the Malaise Inventory which has not been validated as a screening or diagnostic test for mental disorders in general. However, the scale has shown moderate validity against psychiatric morbidity [59] and acceptable psychometric properties [68]. It has been used across population groups, including high risk groups [69, 70] and the abbreviated 9-item subscale has gone on to be used in other cohort studies [71, 72].

Second, the indicators of ACE in the present study were primarily based on measures of socioeconomic position rather than child maltreatment, domestic violence or victimization. Nonetheless, the probability of exposure to these more proximal risk factors is significantly greater for children in low socioeconomic position families [11, 73].

Third, child IQ was derived by proxy from cognitive test scores, however the sensitivity and specificity of our proxy measure is unknown. When using proxy measures of IQ, it is possible that at the tails of the IQ distribution in low functioning individuals may be over or under estimated thus leading to inaccuracies in prevalence rates [74].

Fourth, there has been significant attrition at the survey follow up points. Multiple imputation has been shown to be effective in reducing the bias resulting from missing items when the magnitude of the bias is high and the imputation models are well specified. However, we cannot be certain to have eliminated all sources of bias [75].

### Implications for future research

The findings highlight the complex association between BIF, ACEs and future psychiatric morbidity and the need for further research in this area. Shonkoff, Boyce and McEwen [76] argue that disorders in adulthood often have their origins in disruptions in childhood developmental processes mainly due to biological insults that can manifest many years later. Therefore, study of such processes may encourage earlier intervention with greater certainty than currently exists.

Achievable practical objectives could include: strengthening child protection procedures to reduce risk of exposure to ACEs; increasing awareness and inclusion of questions about intellectual impairment in diagnostic interviews in primary and secondary care; identification of ACEs which should alert health and social care professionals about the potential of the presence of BIF in the index child, leading to follow up assessments or specialist referrals.

It is of concern that the visibility of BIF may decrease further as this condition has been excluded from the new version of the WHO International Classification of Diseases (ICD-11). Paradoxically, though, there is a growing interest on the topic by some public agencies and governments [77]. Our results, clearly accord with previous research that shows the detrimental impact of socio-economic disadvantage mainly in children with intellectual disabilities [78, 79] who are also more likely to experience bullying, neglect and emotional, sexual, physical abuse [78]. Building international consensus on the BIF [80] may not only increase awareness but also encourage development of interventions and the re-examination of its diagnostic utility.

## Conclusions

Whilst low IQ can be seen as an ACE itself, the strength of the study is that it has improved on previous research [81], by the fact that the present longitudinal data were collected prospectively rather than retrospectively. BIF is a little understood entity and often subsumed within a wider “normal” range of ability and functioning. This leads many children, young people and adults to fall outside statutory services and they are, therefore, unable to receive specialist support. By bringing the issue to light, it is our hope that not only we will promote interdisciplinary collaboration and raise awareness but also foster interest in developing preventive and remedial interventions at population and individual level to combat ACEs to improve child and family welfare.

## Data Availability

This is publicly archived dataset based at the Centre for Longitudinal Studies, UCL. Anonymised data are available to download from the UK Data Service. The datasets generated and/or analysed during the current study are available in the https://cls.ucl.ac.uk/cls-studies/1970-british-cohort-study/

## Abbreviations

ACEs: Adverse Childhood Experiences
BCS70: 1970 British Cohort Study
BIF: Borderline intellectual functioning
DSM: Diagnostic and Statistical Manual
ICD: International Classification of Diseases
IQ: Intelligence Quotient
PTSD: Post Traumatic Stress Disorder
